# Field-free deterministic switching of all–van der Waals spin-orbit torque system above room temperature

**DOI:** 10.1126/sciadv.adk8669

**Published:** 2024-03-15

**Authors:** Shivam N. Kajale, Thanh Nguyen, Nguyen Tuan Hung, Mingda Li, Deblina Sarkar

**Affiliations:** ^1^ MIT Media Lab, Massachusetts Institute of Technology, Cambridge, MA 02139, USA.; ^2^ Department of Nuclear Science and Engineering, Massachusetts Institute of Technology, Cambridge, MA 02139, USA.; ^3^ Frontier Research Institute for Interdisciplinary Sciences, Tohoku University, Sendai 980-8578, Japan.

## Abstract

Two-dimensional van der Waals (vdW) magnetic materials hold promise for the development of high-density, energy-efficient spintronic devices for memory and computation. Recent breakthroughs in material discoveries and spin-orbit torque control of vdW ferromagnets have opened a path for integration of vdW magnets in commercial spintronic devices. However, a solution for field-free electric control of perpendicular magnetic anisotropy (PMA) vdW magnets at room temperatures, essential for building compact and thermally stable spintronic devices, is still missing. Here, we report a solution for the field-free, deterministic, and nonvolatile switching of a PMA vdW ferromagnet, Fe_3_GaTe_2_, above room temperature (up to 320 K). We use the unconventional out-of-plane anti-damping torque from an adjacent WTe_2_ layer to enable such switching with a low current density of 2.23 × 10^6^ A cm^−2^. This study exemplifies the efficacy of low-symmetry vdW materials for spin-orbit torque control of vdW ferromagnets and provides an all-vdW solution for the next generation of scalable and energy-efficient spintronic devices.

## Teaser

A van der Waals ferromagnet can now be switched electrically, above room temperature, and without external magnetic fields.

## INTRODUCTION

The discovery of emergent magnetism in two-dimensional (2D) van der Waals (vdW) materials (*1*–*3*) has broadened the material space for developing spintronic devices for energy-efficient, nonvolatile memory and computing applications (*4*–*8*). These applications are particularly well-served by perpendicular magnetic anisotropy (PMA) ferromagnets, which allow fabrication of nanometer scale, high-density, and thermally stable spintronic devices. vdW materials provide strong PMA alternatives (*9*–*12*) to the few optimal bulk material systems, like CoFeB/MgO (*13*–*15*), while providing key advantages like scalability down to monolayer thicknesses, and still maintaining an atomically smooth interface and minimal intermixing with the tunnel barrier of a magnetic tunnel junction (MTJ). The ability to switch a vdW PMA ferromagnet above room temperature is necessary to harness these capabilities for the growing spintronic applications. Hence, recent reports on achieving current controlled switching of vdW PMA ferromagnets at room temperature are promising (*16*, *17*). However, existing schemes for room temperature current control of vdW ferromagnets use spin-orbit torque (SOT) from heavy metals or topological insulators and require the application of an in-plane magnetic field to allow deterministic switching. This creates obstacles in the development of high-density, thermally stable SOT switching devices using vdW ferromagnets. Thus, a solution for field-free, deterministic, and nonvolatile control of PMA magnetism in vdW materials above room temperature is vital for the burgeoning of vdW spintronics.

**Fig. 1.**
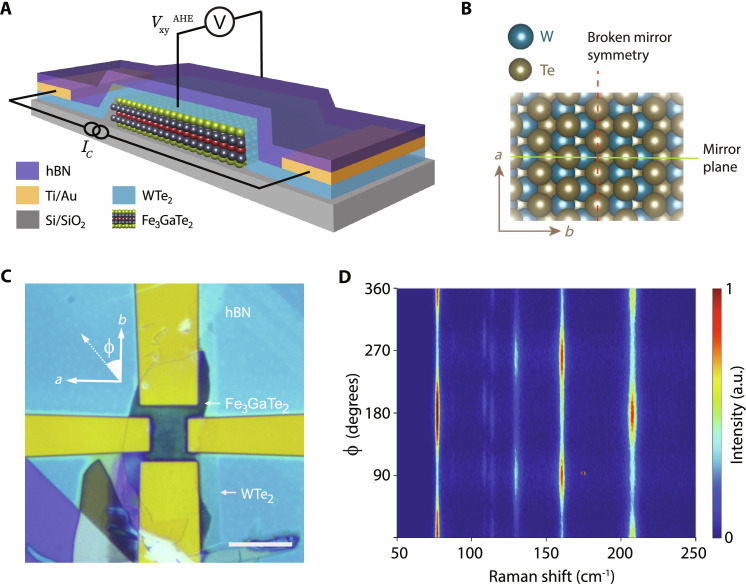
FGaT/WTe_2_ heterostructure device. (**A**) Schematic diagram of the FGaT/WTe_2_ heterostructure devices used in this study. (**B**) Schematic model of WTe_2_ crystal’s *ab* plane, with the *a* and *b* axes labeled. The crystal preserves mirror-plane symmetry in the *bc* plane but breaks it in the *ac* plane. (**C**) Optical image of device D1, with the WTe_2_ (21.6 nm), FGaT (25.8 nm), and hBN flakes labeled. Crystallographic axes of the WTe_2_ flake (determined through polar Raman spectra) and the definition of azimuthal angle ϕ in the Raman spectra are also indicated. Scale bar, 10 μm. (**D**) Polarized Raman spectra of the WTe_2_ flake in (C). The minima (maxima) in type I A_g_ modes at 81 cm^−1^ and 212 cm^−1^ (type II A_g_ mode at 165 cm^−1^) around ϕ= 90° corresponds to the *a* axis of the WTe_2_ flake.

Here, we report the deterministic and nonvolatile switching of a PMA vdW ferromagnet above room temperature without any external magnetic fields. We achieved this by building an all-vdW bilayer SOT system of room temperature PMA vdW ferromagnet, Fe_3_GaTe_2_ (FGaT), with the low-symmetry vdW material WTe_2_ to harness the unconventional out-of-plane anti-damping torque for SOT switching (Fig. 1A). The recently discovered metallic vdW ferromagnet, FGaT, exhibits a record-high intrinsic Curie temperature (*T*_C_, ~350 K) with a strong PMA energy density (*K*_u_ = 3.88 J m^−3^) and has already enabled the creation of the first, all-vdW room temperature MTJs (*10*, *18*). However, a method for electrically controlling its magnetization without magnetic fields is still missing. While several approaches to enabling field-free SOT switching of PMA magnetization are possible, including spin transfer torque-assisted SOT switching (*19*), anisotropy tilting in the ferromagnet (*20*, *21*), artificially breaking lateral symmetry (*22*), and using intrinsically low-symmetry spin-orbit coupling (SOC) layers (*23*–*27*), we have used WTe_2_ because it is particularly interesting for control of vdW magnets, allowing creation of vdW heterostructures and ensuring pristine interfaces and no lattice strain. Charge current injection along the low-symmetry *a* axis of WTe_2_ generates an unconventional, out-of-plane anti-damping SOT, $\tau_{\text{AD}}^{\text{OOP}}$, of the form $\hat{m}\times \hat{z}\times \hat{m}$ ( $\hat{m},\hat{z}$ are unit vectors along ferromagnet magnetization and WTe_2_/ferromagnet interface) (*28*, *29*). This anti-damping-like torque, termed so as it acts against the ferromagnet’s intrinsic Gilbert damping from effective magneto-crystalline anisotropy field, can be used for field-free switching of PMA ferromagnets (*24*–*26*, *30*). Theoretical studies into the microscopic origin of the unconventional torque point to the spin-Hall effect as the major contributing factor (*29*). However, an experimental study on the thickness dependence of SOTs from WTe_2_ has revealed only a small variation in the magnitude of $\tau_{\text{AD}}^{\text{OOP}}$ upon reducing the thickness even down to the monolayer limit (*31*). Previously, WTe_2_ has been used for field-free switching of PMA magnetism in bulk SrRuO_3_ (40 K) (*25*), vdW ferromagnet Fe_3_GeTe_2_ (up to 200 K) (*24*, *27*, *32*), and CoFeB (300 K) (*26*). Thus, this mechanism held promise for achieving the much-anticipated goal of enabling field-free switching of PMA magnetism in a vdW ferromagnet. Using our FGaT/WTe_2_ heterostructure devices, we demonstrate deterministic switching using a low current density up to 320 K. We also show that such field-free deterministic switching is seen exclusively when the charge current is injected parallel to the low-symmetry axis of WTe_2_, asserting the role of crystal symmetry in enabling the field-free switching of PMA magnetism.

## RESULTS

Our heterostructure devices use exfoliated sheets of FGaT and WTe_2_, with patterned electrical contacts, and hexagonal boron nitride (hBN) encapsulation for air stability, as illustrated schematically through Fig. 1A. The heterostructures were assembled using the dry viscoelastic transfer process (*33*), and electrodes were patterned using a combination of e-beam lithography and e-beam evaporation of Ti/Au (more details in Materials and Methods). The T_d_ phase of WTe_2_ used here belongs to the Pmn2_1_ space group. As shown in Fig. 1B, the crystal structure of WTe_2_ is such that it preserves mirror symmetry about the *bc* plane (σ*_bc_*), while it breaks the mirror symmetry along the *ac* plane (σ*_ac_*), where *c* is the out-of-plane crystallographic axis. As a result, SOC induced spin accumulation, and consequently, the SOT, in response to a current flowing along the *a* axis and the *b* axis, varies substantially. These two cases are treated in detail in the following discussion, using two devices, D1 with FGaT (25.8 nm)/WTe_2_ (21.6 nm) and D2 with FGaT (17.9 nm)/WTe_2_ (23.8 nm). An optical image of the device D1 is shown in Fig. 1C, with the FGaT, WTe_2_, and hBN flakes indicated. The crystallographic *a* and *b* axes of the WTe_2_ flakes were identified using polarized Raman spectroscopy in the backscattering geometry $Z\left(\hat{\phi }\hat{\phi }\right)\bar{Z}$ , where $\hat{\phi }$ is a unit vector in the sample plane, along the azimuthal angle ϕ as defined in Fig. 1C. Figure 1D shows a color plot of the polarized Raman spectra of the WTe_2_ flake in D1 (see fig. S2 for D2). WTe_2_ exhibits two types of prominent A_g_ peaks with twofold symmetries, which can be used to identify its crystallographic *a* and *b* axes (*34*, *35*). The minima in the type I peaks (81 and 212 cm^−1^), which coincide with the maxima in the type II peak (165 cm^−1^), correspond to the *a* axis of the WTe_2_ crystal.

**Fig. 2.**
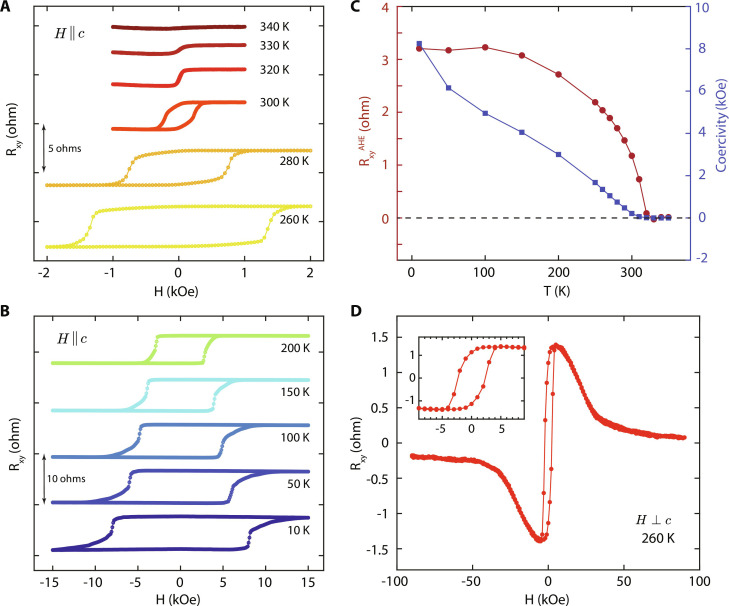
Magneto-transport characterization of FGaT/WTe_2_ devices. (**A** and **B**) Anomalous Hall effect (AHE) measurements for field swept out of the sample plane (**H** ∥ *c*) at varying temperatures up to 340 K. Data presented corresponds to device D1 (Fig. 1C). Data are offset along *y* axis for clarity. (**C**) Variation of anomalous Hall resistance ( $R_{\text{xy}}^{\text{AHE}}$ , left *y* axis) and coercivity (*H*_c_, right *y* axis) with temperature. (**D**) Anomalous Hall effect measurement for field swept close to the sample plane (**H** ⊥ *c*), with transverse resistance reaching near-zero level at high fields, indicative of the strong perpendicular magnetic anisotropy of FGaT being preserved in the FGaT/WTe_2_ heterostructure devices. Inset: Detailed view of the AHE curve at low fields.

Magneto-transport characterization of the FGaT/WTe_2_ devices using anomalous Hall effect helps to establish that the inherent ferromagnetic characteristics of FGaT are well preserved in the heterostructure device and can be effectively probed through transverse voltage monitoring for current-induced magnetization switching experiments. Figure 2 (A and B) shows the anomalous Hall effect curves for the device D1, for field swept along sample normal (**H** ∥ *c*) and temperatures in the range 10 to 340 K. The device exhibits a large coercivity (up to 8.25 kOe at 10 K) at low temperatures, which diminishes with temperature (Fig. 2C) such that *H*_c_ = 210 Oe at 300 K and near zero starting 330 K. The anomalous Hall resistance, $R_{\text{xy}}^{\text{AHE}}$ goes to zero above 320 K too, marking a ferromagnet to paramagnet transition between 320 and 330 K (Fig. 2C). The anomalous Hall effect curve corresponding to field swept close to the sample plane (**H** ⊥ *c*) is shown in Fig. 2D. It exhibits the characteristics of a PMA magnet, going to near-zero resistance values only at high in-plane magnetic fields, with an effective anisotropy field of about 35 kOe, corroborating that the strong PMA of FGaT is preserved in the heterostructure device.

**Fig. 3.**
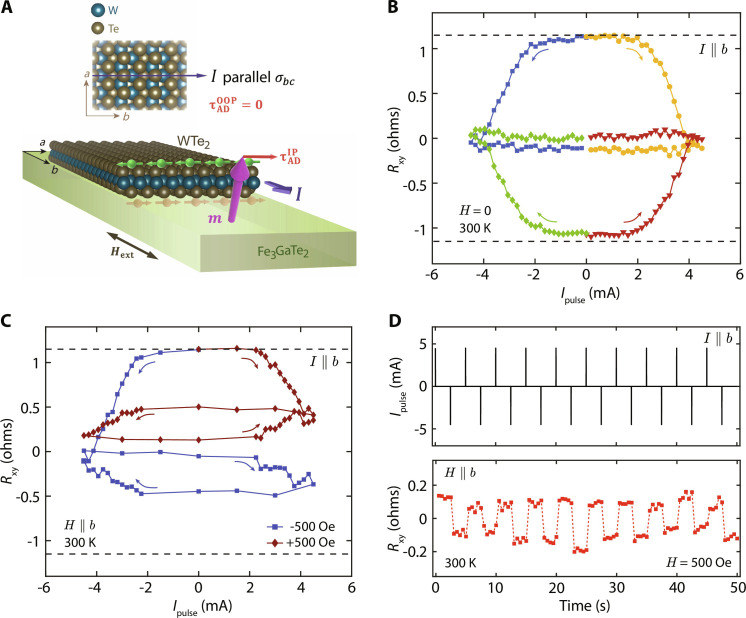
Field-assisted (only) switching for **I** **||** *b*. (**A**) Schematic illustration of the scenario where current is sourced along the high-symmetry axis, *I* ∥ *b*. Symmetry constraints allow only an in-plane component of spin accumulation along the FGaT/WTe_2_ interface, resulting in a nonzero in-plane anti-damping torque ( $\tau_{\text{AD}}^{\text{IP}}\neq 0$ ) but a zero out-of-plane anti-damping torque ( $\tau_{\text{AD}}^{\text{OOP}}=0$ ). (**B**) Response of the device to current pulses applied along the *b* axis at zero external field at 300 K. The blue and green (yellow and red) curves correspond to current pulses swept from 0 →−4.5 mA → 0 (0 →+4.5 mA → 0 mA), for the device initialized at **m**_**z**_ = 1 and **m**_**z**_ = −1, respectively. The device undergoes complete demagnetization by 4.5 mA in all the four cases. (**C**) Current sweeps up to 4.5 mA result in partial magnetization switching in the presence of an externally applied field, **H** ∥ *b* ∥ *I* of ±500 Oe, with changing the direction of field resulting in chirality reversal of the current-induced switching curves. Black dashed lines in (B) and (C) correspond to **m**_**z**_ = ±1. (**D**) Field-assisted deterministic, nonvolatile switching of FGaT magnetization using a train of 1-ms-long current pulses, ±4.5 mA in magnitude, under +500 Oe in-plane magnetic field, **H** ∥ *b* ∥ *I*.

Figure 3A provides a schematic representation of the SOT mechanism at play when the applied current is parallel to the high-symmetry *b* axis. In this case, the applied current has no effect on the crystal’s *bc*-mirror plane symmetry (σ*_bc_*). In accordance with Curie’s principle (*36*), since the causalities (crystal structure and applied current) preserve σ*_bc_*, the resultant spin current (and accumulation) must also preserve σ*_bc_*. This forbids a vertical spin-polarization (**σ***_**z**_*) component in the vertically flowing spin current, since the **σ***_**z**_* pseudovector transforms anti-symmetrically upon reflection in the *bc* plane. As a result, the spin accumulation at the FGaT/WTe_2_ interface only has an in-plane spin-polarization, similar to the case of typical heavy metal/ferromagnet and topological insulator/ferromagnet systems. Such an in-plane spin accumulation can only produce deterministic switching in the presence of an externally applied field along the current direction. Figure 3B shows the response of device D1 to current pulses applied along the *b* axis of WTe_2_ in the absence of any external field, at 300 K. As expected, the in-plane anti-damping torque from spin accumulation at the FGaT/WTe_2_ interface drives the FGaT magnetization in-plane (**m**_**z**_ = 0), resulting in a near-zero anomalous Hall resistance, for a current magnitude of about ±4.5 mA (9.51 × 10^5^ A cm^−2^). Upon lowering the current drive to zero, the FGaT remains effectively demagnetized as its various domains orient randomly due to lack of a symmetry breaking field. The four curves in Fig. 3B verify this for all combinations of current drives (positive or negative) and initial magnetization directions (**m**_**z**_ = ±1 ≡*R*_xy_ = ±1.2 ohms). The initial magnetization state is set by applying a field of ±2 kOe along the sample normal before starting current sweeps. Contrary to the above case, driving a current of the same magnitude in the presence of a nonzero external field (**H** = ±500 Oe) parallel to current axis, **H** ∥ *I* ∥ *b*, results in deterministic, partial switching of the FGaT magnetization. As shown in Fig. 3C, reversing the direction of applied field reverses the chirality of the current-induced switching loops, as is expected for such a system. Field-assisted deterministic and nonvolatile switching of out-of-plane magnetization of FGaT could also be achieved in D1 for the case of **H** ∥ *I* ∥ *b* (Fig. 3D) at 300 K.

**Fig. 4.**
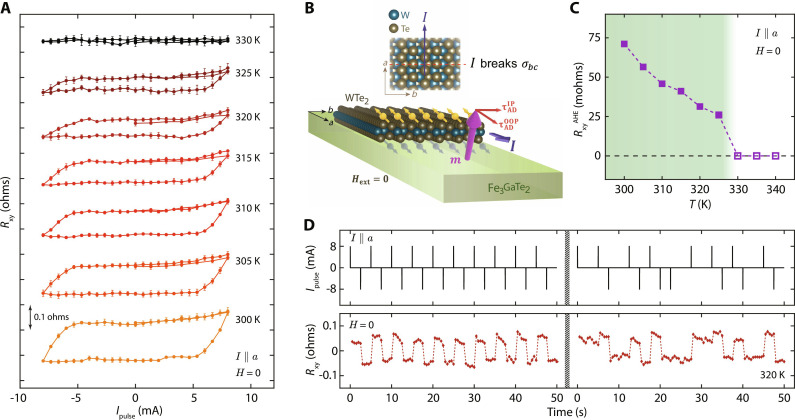
Field-free switching for *I* **||** *a*. (**A**) Response of the device D2 to current pulse sweeps, along *a* axis, for varying temperatures without any external field. The curve at each temperature is an average of four consecutive current pulse sweeps acquired for that temperature, with error bars indicating SD of each data point across the four sweeps (individual sweeps reported in fig. S5). Data offset along *y* axis for clarity. (**B**) Schematic illustration of this scenario where current is sourced along the low-symmetry axis, *I* ∥ *a*. Broken mirror plane symmetries allow an out-of-plane component of spin accumulation along the FGaT/WTe_2_ interface, resulting in a nonzero out-of-plane anti-damping torque ( $\tau_{\text{AD}}^{\text{OOP}}\neq 0$ ), which is asymmetric in current direction, enabling field-free deterministic switching of the underlying FGaT’s magnetization. (**C**) Temperature dependence of anomalous Hall resistance splitting in the current-induced switching loops. Clear switching can be observed up to 325 K (green region), with decreasing $R_{\text{xy}}^{\text{AHE}}$ , as denoted with solid square points, while no clear switching loops could be observed starting 330 K (white region), and hence, $R_{\text{xy}}^{\text{AHE}}$ is set to zero (hollow square points). (**D**) Demonstration of field-free, deterministic, and nonvolatile switching of out-of-plane FGaT magnetization in the FGaT/WTe_2_ device using 1-ms-long pulses of current, ±8 mA in amplitude, applied along the *a* axis. The data are acquired at 320 K in two sets of 50-s-long pulsing sequences, with periodic and randomized current pulses, respectively.

In contrast to the above discussed case, when current is applied along the low-symmetry *a* axis of WTe_2_, the applied current breaks the *bc*-mirror plane symmetry (σ*_bc_*). Thus, the causalities break both the mirror plane symmetries (σ*_ac_* broken by crystal structure, σ*_bc_* broken by applied current), and a vertical spin-polarization component in the vertical spin current is now permissible. This scenario is depicted schematically in Fig. 4B. The vertical component of spin accumulation at the FGaT/WTe_2_ interface can now apply a symmetry breaking, unconventional, out-of-plane anti-damping spin-orbit torque, $\tau_{\text{AD}}^{\text{OOP}}$ , on the FGaT magnetization. $\tau_{\text{AD}}^{\text{OOP}}$ is anti-symmetric in current, and hence, the FGaT magnetization can be toggled deterministically between **m**_**z**_ = ±1 by applying positive and negative current pulses. Device D2, with current applied along the *a* axis of its WTe_2_ flake, is used to study this scenario. Details of the device, including its Raman spectra and magneto-transport data, are presented in figs. S2 and S3. Figure 4A shows the field-free current induced switching loops of D2 for temperatures ranging from 300 to 325 K. At 300 K, maximum switching could be induced using ±8 mA (see fig. S4), equivalent to a current density of 2.23 × 10^6^ A cm^−2^. Increasing the temperature from 300 to 325 K resulted in shrinking of the anomalous Hall resistance splitting, until no clear looping behavior could be observed at 330 K and beyond (Fig. 4C). This aligns with the fact that magnetization of FGaT would decrease with increasing temperature, resulting in a decreasing $R_{\text{xy}}^{\text{AHE}}$ until it eventually vanishes beyond its *T*_C_ (320 to 330 K). Figure 4D shows the response of the device to a train of current pulses, 1 ms long and ±8 mA in amplitude, applied along the low-symmetry axis of WTe_2_, *I* ∥ *a*, at 320 K. Deterministic and nonvolatile switching of the transverse resistance in response to the current pulses ratifies the robustness of the proposed switching scheme. Similar switching data for the device at 300 and 310 K are presented in fig. S6.

## DISCUSSION

We use the unconventional, out-of-plane anti-damping spin-orbit torque, $\tau_{\text{AD}}^{\text{OOP}}$, generated from WTe_2_ upon charge current injection along its low-symmetry *a* axis to switch the magnetization of underlying FGaT in the FGaT/WTe_2_ heterostructure devices. We show that the $\tau_{\text{AD}}^{\text{OOP}}$ induced field-free switching occurs exclusively for charge current injection along WTe_2_ *a* axis, while charge injection along the *b* axis results in demagnetization of underlying FGaT. We observe the field-free switching up to 320 K, determined by the Curie temperature of our FGaT crystals (320 to 330 K). It would be interesting to explore substrate engineering to push FGaT’s Curie temperature even further, as has successfully been attempted in similar materials. For example, the intrinsic *T*_C_ of Fe_3_GeTe_2_ is ~210 K but it increased to 400 K when grown epitaxially on Bi_2_Te_3_ (*37*). Similarly, the intrinsic *T*_C_ of Fe_4_GeTe_2_ is ~270 K and it increased to 530 K upon epitaxial growth on Al_2_O_3_ (*38*). Recently, it has also been shown that T_d_-TaIrTe_4_, another low-symmetry vdW material isomorphic to WTe_2_ wherein W atoms are replaced by alternating dimers of Ta-Ta and Ir-Ir, can also induce field-free deterministic switching in PMA CoFeB (*39*, *40*). This further reinforces the potential of low-symmetry vdW materials for such applications and provides a direction for material exploration to get more efficient SOT systems.

While FGaT checks several of the boxes for an ideal ferromagnet for vdW spintronics, including metallicity, PMA, above room temperature *T*_C_, and a strong magnetic anisotropy energy (MAE) (*K*_u_ = 3.88 J m^−3^) (*10*), several more developments are needed to allow its translation to commercial spintronic devices. The magnetic properties of the material in the 2D limit (monolayers) are yet to be studied. It is commonly seen that the *T*_C_ of a ferromagnet decreases upon thinning it down to a few atomic layers (*2**,* *41**,* *42*). Interestingly though, our density functional theory (DFT) calculations reveal that the MAE of FGaT monolayer is about 25% greater than its bulk value (details in fig. S7 and table S1). This is a promising result as the stronger PMA can help stabilize the monolayer (2D) Ising system up to a higher temperature and can act as a counterweight to the dimensionality effects to ensure above room temperature operation even in the monolayer limit. It should also be noted that the devices reported herein are several micrometers in lateral dimensions. The performance of the devices can be expected to vary upon reducing lateral dimensions to sub–100 nm scale where low threshold switching processes like domain wall propagation are less prevalent. Finally, an important milestone toward enabling scalable fabrication of such devices is the wafer scale growth of few-layer FGaT, as has been demonstrated for similar materials previously (*38**,* *43*).

In conclusion, we have reported the first demonstration of field-free magnetization switching of a PMA vdW ferromagnet above room temperature (up to 320 K) using a low current density of 2.23 × 10^6^ A cm^−2^. The proposed all-vdW architecture can provide unique advantages like improved interface quality needed for efficient SOTs, possibilities for gate-voltage tuning to assist SOT switching, and prospects for flexible and transparent spintronic technologies. This work asserts the role of crystal symmetry in SOC layers of an SOT switching device using a low-symmetry vdW material, and provides a new, scalable all-vdW approach to developing energy-efficient spintronic devices.

## MATERIALS AND METHODS

### Synthesis of FGaT bulk crystals

The bulk crystals of FGaT were synthesized using a flux method with a molar ratio of 1:2:2 of Fe powder (Beantown Chemical, 99.9%), Ga ingot (Alfa Aesar, 99.99999%), and Te pieces (Sigma-Aldrich, 99.999%). These constituents were handled and thoroughly mixed in a N_2_-filled glovebox, with H_2_O and O_2_ levels less than 0.1 ppm. These were subsequently placed in a Canfield-type crucible and flame-sealed in an evacuated quartz tube. The mixture was heated to 1000°C in an hour and dwelled at that temperature for 24 hours. This was followed by a fast-cooling step to 880°C in 1 hour and then a slow-cooling step down to 780°C in 100 hours, after which centrifugation was performed to remove the excess Te flux. The crystals were subsequently heat-treated and were extracted from the opened ampules in the inert environment of a N_2_-filled glovebox. The crystals have a morphology of thin millimeter-sized platelets with a metallic luster.

### Device fabrication

The FGaT/WTe_2_ devices reported here were fabricated using heterostructure assembly of exfoliated vdW flakes. Bulk FGaT was grown using a flux method as discussed above. Bulk WTe_2_ and hBN were commercially sourced from HQ Graphene and Ossila, respectively. FGaT flakes were exfoliated on Si/SiO_2_ (280 nm) substrates using mechanical exfoliation. WTe_2_ flakes, exfoliated on polydimethylsiloxane stamps, were transferred on to selected FGaT flakes using the dry viscoelastic transfer process. Electrodes were then patterned on the FGaT/WTe_2_ heterostructure using a combination of e-beam lithography with the positive e-beam resist PMMA 950 and e-beam evaporation of Ti/Au (5 nm/60 nm). The devices were then encapsulated with thick exfoliated flakes of hBN using dry viscoelastic transfer. All exfoliation and vdW transfer processes were performed inside the inert environment of a N_2_-filled glovebox (O_2_, H_2_O < 0.01 ppm). Thicknesses of the constituent flakes were characterized after encapsulation using a Cypher VRS AFM. Polarized Raman spectra of WTe_2_ flakes were acquired using a 532-nm laser with a WITec Alpha300 Apyron Confocal Raman microscope, by rotating the polarizer and analyzer while the sample was static.

### Transport measurements

All transport measurements were performed in a 9 T PPMS DynaCool system. Measurements were performed by sourcing current using a Keithley 6221 current source and measuring the transverse voltage across the devices, using a Keithley 2182A nanovoltmeter. Anomalous Hall effect measurements with field sweeps were performed using a drive current of 50 to 200 μA. For the current-induced switching measurements, a 1-ms pulse of write current was followed by 999 ms of read pulses (±200 μA). Field could be applied in and out of the sample plane using the PPMS horizontal rotator module.

### First-principles calculations

Electronic property calculation and structural optimizations were performed using DFT with the Quantum ESPRESSO package (*44**,* *45*). We use the optimized norm-conserving Vanderbilt (ONCV) pseudopotentials (*46*) with Perdew-Burke-Ernzerhof (PBE) functionals (*47*) to account for the exchange-correlation interaction. To accurately describe the structural properties of layered FGaT structure, we use the nonlocal vdW-DF2 functional (*48*) for the vdW interaction. A large plane wave cutoff energy of 60 Ry (~816 eV) is used for all calculations. The FGaT monolayer and bilayer are modeled by the slab supercells, with the separations between the neighboring slabs being about 20 Å. A 16 × 16 × 1 *k*-point mesh is used for monolayer and bilayer FGaT, while 14 × 14 × 2 mesh is used for the bulk FGaT. These parameters are selected based on the convergence test of the total energy. The atomic positions and lattice constants are optimized by the Broyden–Fletcher–Goldfarb–Shanno quasi-newton algorithm (*45*), in which the convergence values for the forces and stress components are 0.0001 Ry/a.u.^3^ and 0.005 GPa, respectively. The optimized lattice constants of the FGaT monolayer, bilayer, and bulk are listed in table S1. To determine the MAE of FGaT, we first perform the total energy calculations for an in-plane magnetization (along the *x* axis) and then out-of-plane magnetization (along the *z* axis), including SOC. Then, the MAE is given by the difference in total energy for the two systems, i.e. $\Delta E=E_{\text{SOC}}^{m∥x}-E_{\text{SOC}}^{m∥z}$ (*49*).
