# Supplementary material for: Field-free deterministic switching of all–van der Waals spin-orbit torque system above room temperature
Source: Sci Adv. 2024 Mar 15;10(11):eadk8669. doi: 10.1126/sciadv.adk8669 (PMC10942109; doi:10.1126/sciadv.adk8669)
Supplement: Supplementary file 1 — Figs. S1 to S7 Table S1 [file sciadv.adk8669_sm.pdf]

Science Advances
AAAS

Supplementary Materials for

Field-free deterministic switching of all–van der Waals spin-orbit torque system above room temperature

Shivam N. Kajale *et al.*

Corresponding author: Deblina Sarkar, deblina@mit.edu

Sci. Adv. **10**, eadk8669 (2024)
DOI: 10.1126/sciadv.adk8669

This PDF file includes:

Figs. S1 to S7
Table S1

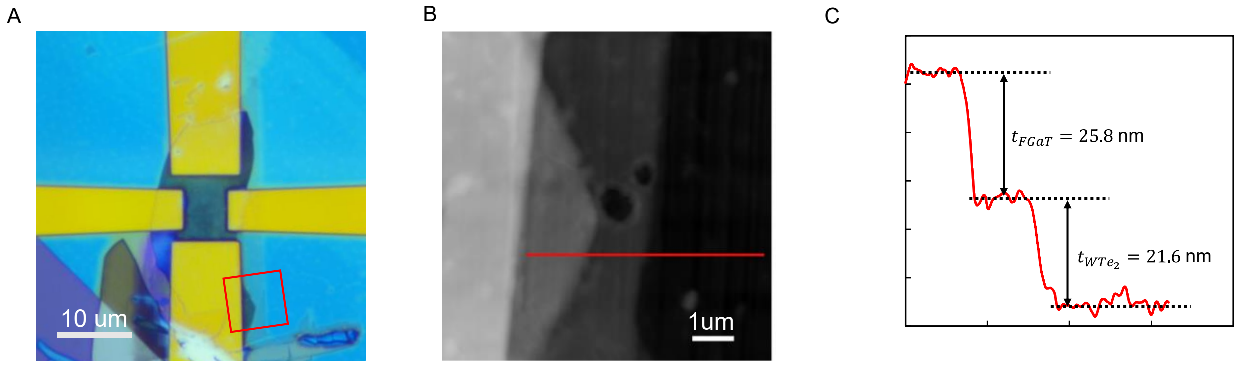

Fig. S1. Topographical data for device D1. (**A**) Optical image of the device, with red box indicating the region used for AFM measurements. (**B**) AFM topography micrograph of the region in red box. (**C**) Height profile along the red line in panel B.

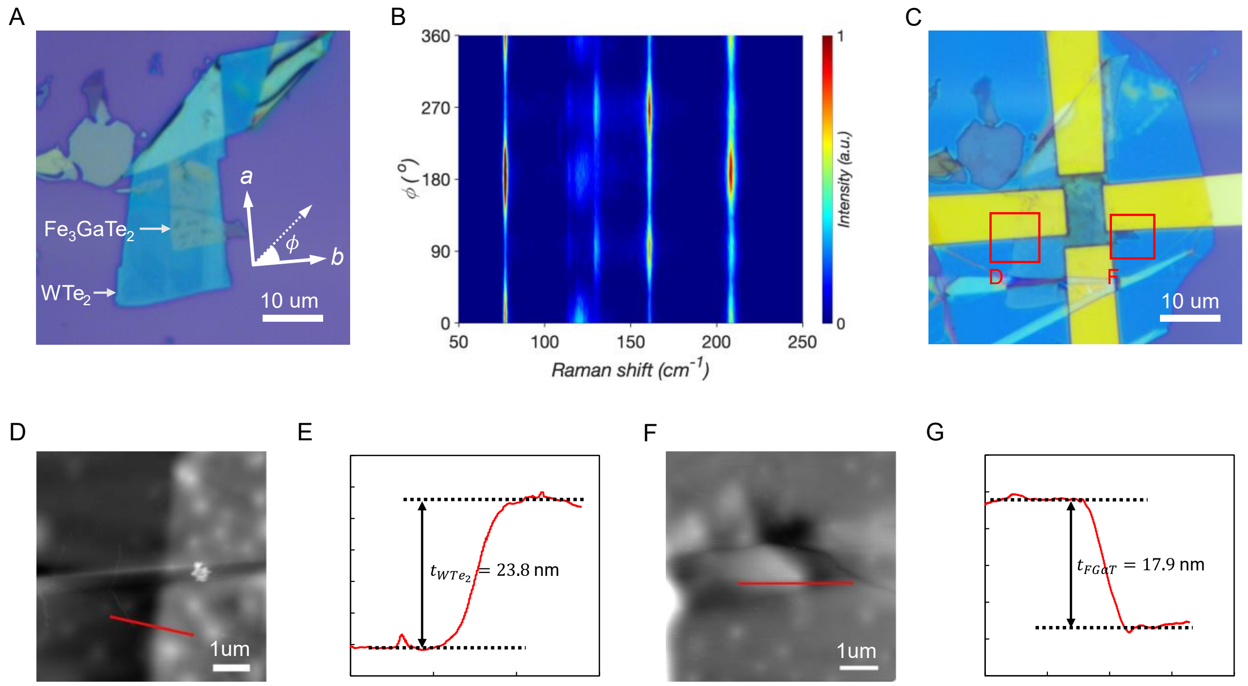

Fig. S2. Device D2, its Raman spectra and topography. (**A**) Optical image of the FGaT/WTe2 heterostructure before patterning electrodes. The crystallographic axes of WTe_2 (determined using polar Raman spectra) and the definition of azimuthal angle ϕ in the polar Raman measurements is indicated. (**B**) Polarized Raman spectra of the WTe_2 flake in D2. (**C**) Optical image of the device D2, after patterning electrodes and encapsulation with hBN. Red boxes correspond to area scanned in AFM for determining the thicknesses of the constituent WTe_2 (box D) and FGaT (box F) flakes. (**D**) AFM topography micrograph of red box D (in panel C) and (**E**) the height profile along the red line (in panel D). (**F**) AFM topography micrograph of the red box F (in panel C) and (**G**) the height profile along the red line (in panel F).

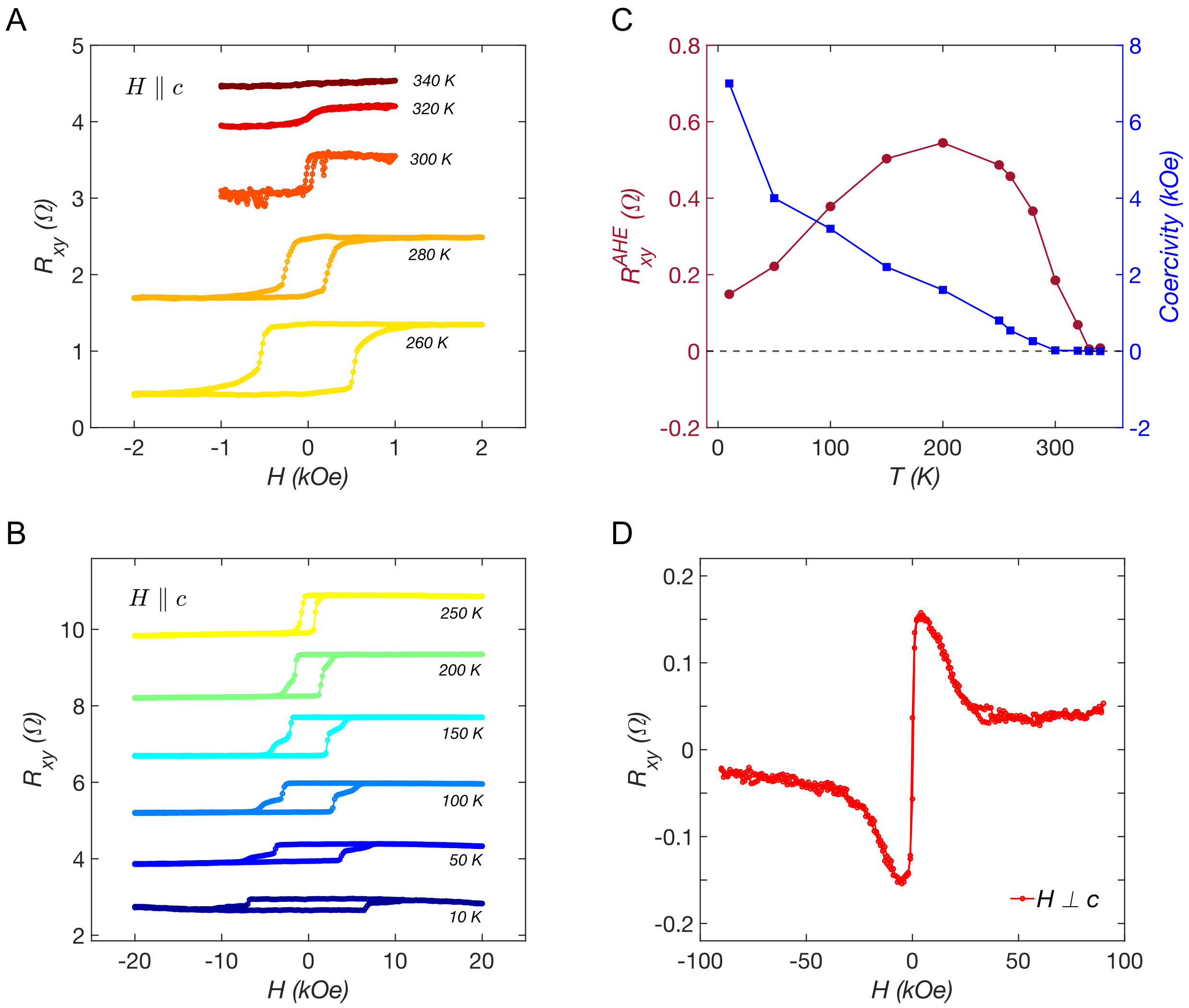

Fig. S3. Magneto-transport characterization of D2. (**A, B**) Anomalous Hall effect measurements for field swept out of the sample plane (**H** ∥ c) for varying temperatures up to 340 K. Data is offset along y-axis for clarity. (**C**) Variation of anomalous Hall resistance (R_{xy}^{AHE}, left y-axis) and coercivity (H_c, right y-axis) with temperature. (**D**) Anomalous Hall effect measurement for field swept close to sample plane (**H** ⊥ c), indicative of the strong perpendicular magnetic anisotropy of FGaT being preserved in the FGaT/WTe_2 heterostructure device.

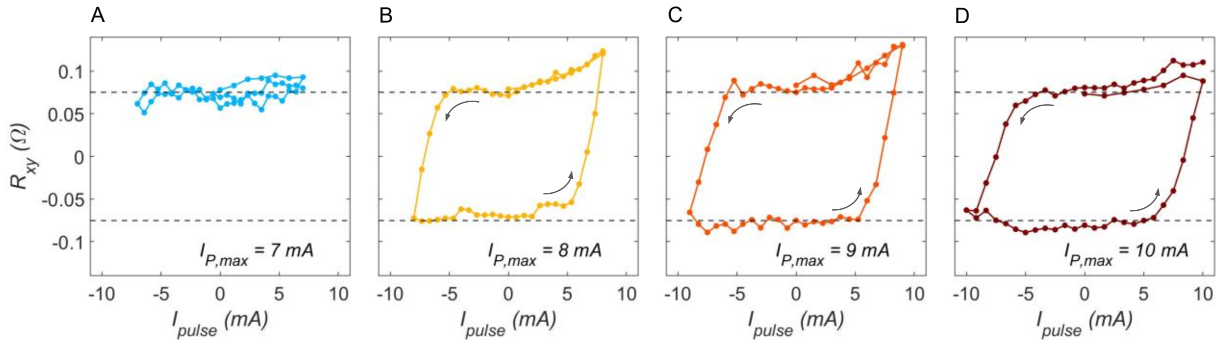

Fig. S4. Effect of increasing peak current in current sweeps in D2. (**A**) No clear switching behavior observed when current sweep is limited to a maximum pulse amplitude $I_{\mathrm{P,max}}$ of ± 7 mA. (**B**) A clear, cyclic switching curve is observed on increasing $I_{\mathrm{P,max}}$ to ± 8 mA. Further increasing $I_{\mathrm{P,max}}$ to (**C**) ± 9 mA and (**D**) ± 10 mA does not increase the loop's vertical splitting notably. Thus, switching is deemed to be near-complete by 8 mA. Black dashed lines are a visual aid denoting the same loop splitting.

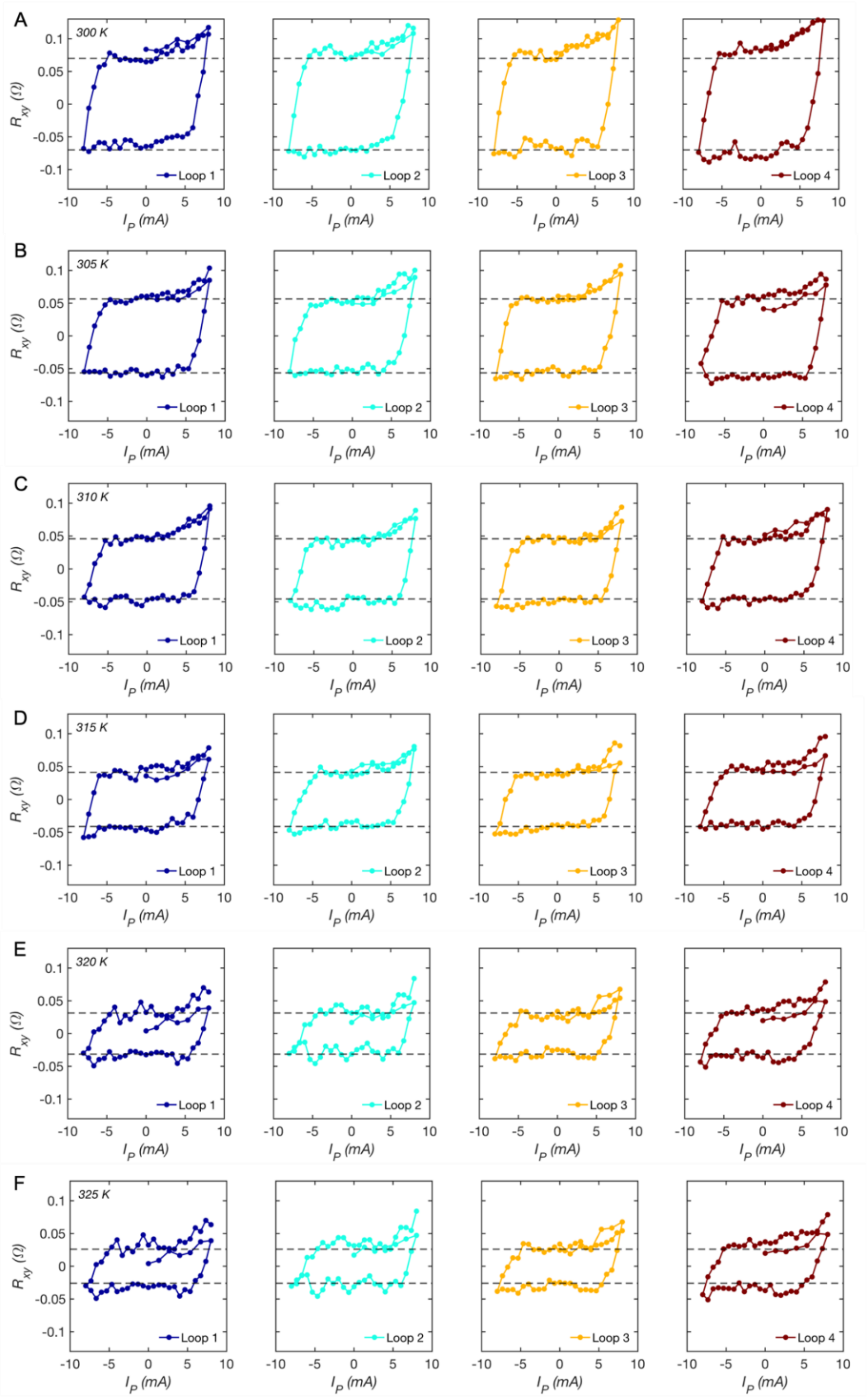

Fig. S5. Current-pulsing loops across varying temperatures. (**A**) Four consecutive current pulsing loops acquired for D2, with $I \parallel a$, without any external field (no field-assisted initialization between consecutive loops either) at 300 K. Black dashed lines are a visual aid denoting the same loop splitting. (**B-F**) Similar data for temperatures 305 K – 325 K in steps of 5 K.

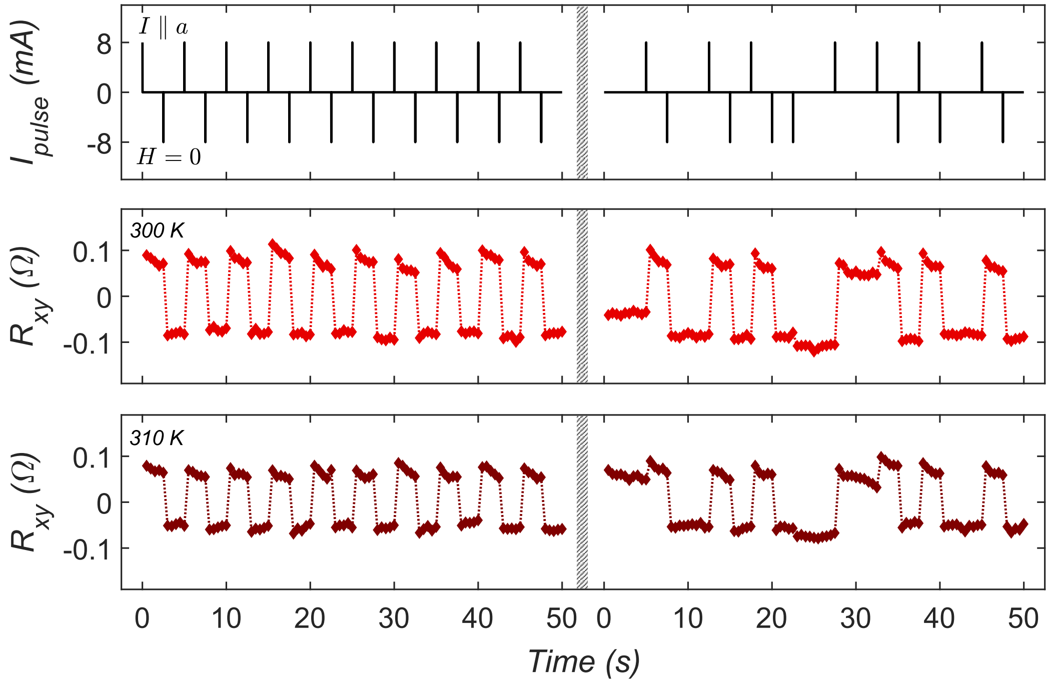

Fig. S6. Deterministic switching at 300 K and 310 K. Field free deterministic, non-volatile switching of the OOP magnetization of FGaT in device D2, using the train of current pulses, 1 ms long and ±8 mA in magnitude (top panel), with $I \parallel a$, at 300 K (middle panel) and 310 K (lower panel). The data is acquired in two sets of 50 s long pulsing sequences, with periodic and randomized current pulses, respectively.

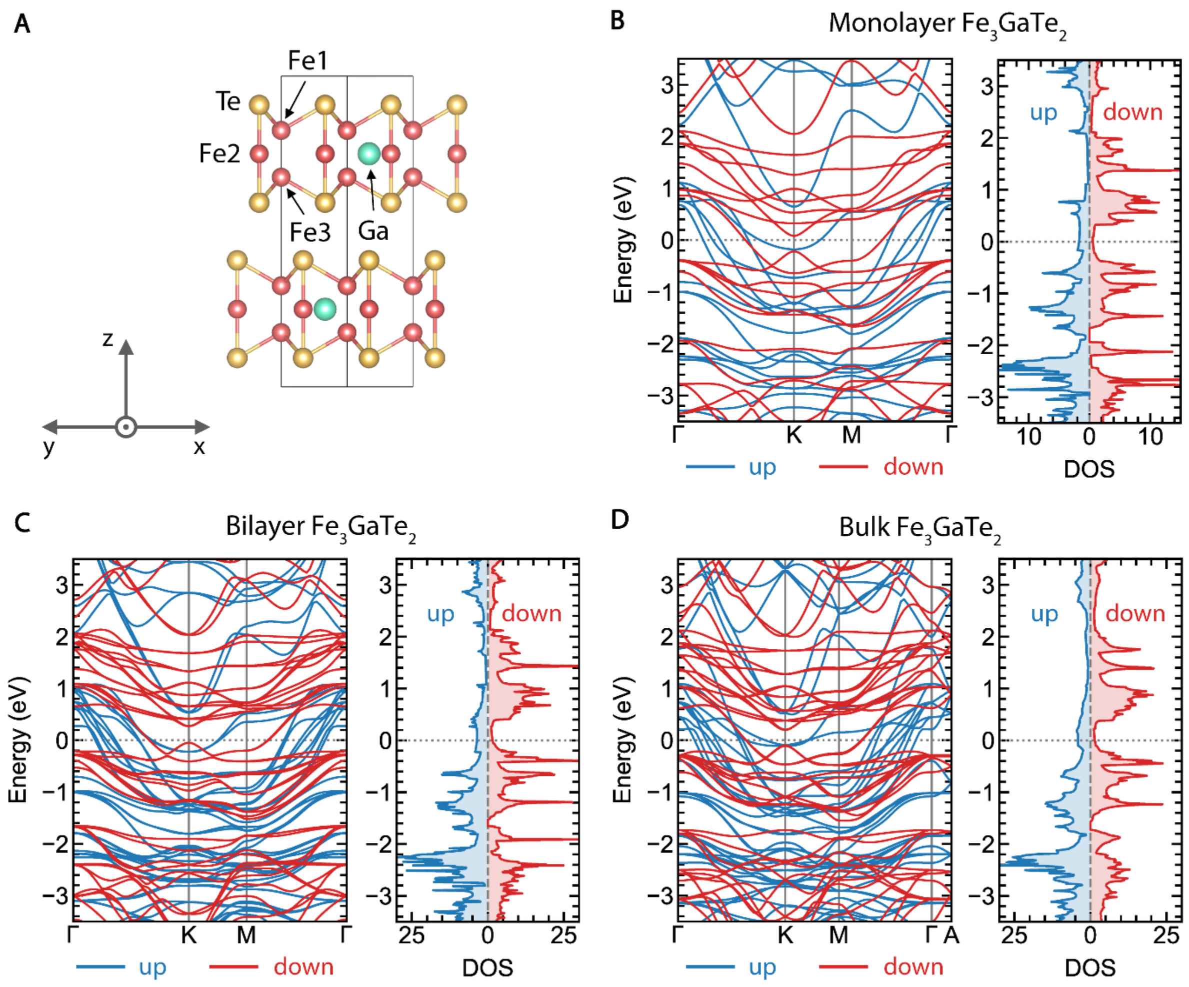

Fig. S7. Thickness dependent DFT calculations for FGaT. (**A**) Schematic illustration of the side view of bilayer FGaT, with different atoms in the crystal annotated. Spin-resolved band structure and density of states calculated for (**B**) monolayer, (**C**) bilayer and (**D**) bulk FGaT. Details of the calculations are included in Materials and Methods.

	a (Å)	c (Å)	Moment on Fe1 (μ_B)	Moment on Fe2 (μ_B)	Moment on Fe3 (μ_B)	Total magnetic moment (μ_B)	Magnetic anisotropy energy (meV)
Monolayer	3.954	-	2.2654	1.4307	2.2654	5.8047	0.254
Bilayer	4.007	-	2.3312	1.5527	2.3312	6.0867	0.186
Bulk	4.023	16.418	2.3317	1.5352	2.3317	6.0070	0.205

Table S1. Thickness dependent magnetic properties of FGaT. Lattice relaxed lattice constants, magnetic moment of constituent Fe atoms (as defined in Fig. S7) and magnetic anisotropy energy of monolayer, bilayer and bulk FGaT, as determined using density functional theory calculations.
